# Airway Papillomatosis: New Treatments for an Old Challenge

**DOI:** 10.3389/fped.2019.00383

**Published:** 2019-09-18

**Authors:** Nankee Kumar, Diego Preciado

**Affiliations:** ^1^Sheikh Zayed Center for Pediatric Surgical Innovation, Children's National Health System, Washington, DC, United States; ^2^Division of Pediatric Otolaryngology, Children's National Health System, Washington, DC, United States

**Keywords:** RRP, bevacizumab, HPV vaccine, cidofovir, interferon-α, indole-3-carbinol

## Abstract

Recurrent respiratory papillomatosis (RRP) is the recurrent growth of small, benign tumors, or papillomas, in the respiratory tract, caused by human papillomavirus (HPV). Currently, there is no cure. Palliative treatments seek to prevent airway obstruction, keep underlying tissues healthy, and maintain voice quality. The most common intervention, the local surgical removal of papillomas, may be inadequate as a standalone treatment for pediatric populations that experience rapid papilloma regrowth, as repeated surgeries cause increased damage to the surrounding tissues and impose significant emotional and economic burden on families. Interferon α and Cidofovir have been shown to lengthen the time between surgical interventions and/or decrease the total number of procedures needed, although the evidence of their efficacy and safety is controversial. Novel therapies, including photodynamic therapy, indole-3-carbinol, anti-reflux medication, heat shock protein, and Mumps and HPV vaccination, may provide potential avenues for treatment, but require further research. Among all the novel therapies investigated, systemic bevacizumab seems to offer the most promising alternative to surgery. Randomized control trials to investigate its impact, especially in a pediatric population, should be conducted before implementing it as a standard form of care. This review will summarize the latest literature on medical care for aggressive RRP disease.

## Introduction

**Figure 1:**
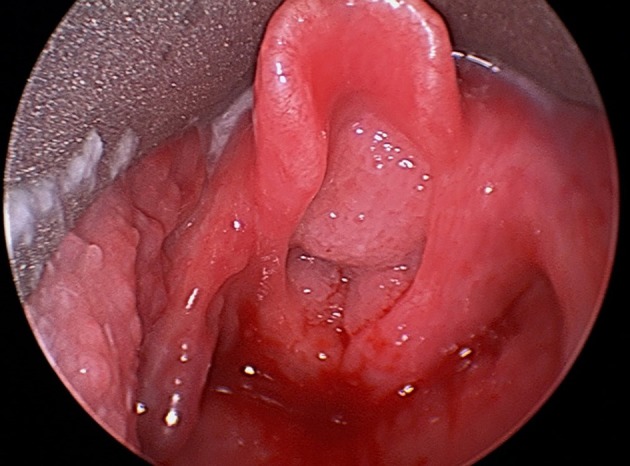
Direct laryngoscopic image of large bulky airway papillomatosis obstructing the laryngeal inlet.

Recurrent respiratory papillomatosis (RRP) is a chronic disease caused by human papillomavirus (HPV), usually types 6 and 11. The lesions of RRP most frequently appear in the larynx (Figure 1), but may also emerge in the mouth, trachea, bronchia, lung parenchyma, and esophagus. The juvenile-onset form of RRP, in which symptoms present before 12 years, is more aggressive than the adult-onset form, and symptoms are more severe. Papilloma recurrence is especially rapid in children under 3 years, who experience shorter intervals between surgical interventions and thus more surgeries overall. The likelihood of the infection spreading beyond the larynx and incidence of a tracheostomy, to help keep the trachea open are also greater in this population (1). Patients with juvenile onset RRP undergo on average 20 surgical interventions, most of which are in childhood (2).

The traditional surgical method for surgical extirpation classically involved KTP (potassium titanyl phosphate) or carbon dioxide lasers (3). Given the potentially severe side effects of laser ablation, including respiratory tract burns and laryngeal scarring, microdebriders have become an increasingly popular surgical tool, particularly among pediatric otolaryngologists, for bulky disease (3). This hand-held device can remove the diseased tissue more precisely, reducing damage to surrounding tissues (3). However, given the aggressive nature of juvenile RRP, debridements are still frequent, and over time, still wear down the underlying tissue significantly, with high incidence of scar formation. As such, several medical therapies have been investigated to improve the outcome of surgery, by increasing the length of time between operations, or by minimizing recurrence of RRP lesions.

## Interferon α

Interferon α, a protein produced by leucocytes in reaction to diverse stimuli, including viral infection, was one of the first potential medical treatments studied (4). Its value as an adjuvant treatment is controversial. In a large study of 85 children and 84 adults, 58% of children ended the year-long treatment period of intramuscular interferon α injections three times a week without evidence of RRP (5). The frequency of papilloma recurrence decreased in 74% of all children treated (5). In a randomized control trial of 123 patients under 21 however, the slowed growth rate of papillomas in the experimental group that received interferon α intramuscular injections was not sustained over the course of the year-long treatment (6). Similarly another study found that, 20 years after the conclusion of treatment with interferon α, only 42% of patients indicated a long term response, and all those who relapsed had the juvenile onset form of RRP (7). Most studies investigating interferon α have also reported serious side effects, like neurologic disorders, leukopenia, and thrombocytopenia (5). For these reasons, its use by pediatric otolaryngologists has dwindled and is generally not recommended (1). In 2014, only 4% of children with RRP received interferon α as adjuvant therapy (1).

## Cidofovir

This broad-spectrum, antiviral drug that inhibits viral DNA polymerases has been shown to be promising as an adjuvant intralesional therapy to surgery for RRP. Intralesional cidofovir injections are the most commonly administered medical treatment for RRP among pediatric otolaryngologists (1). All papers published between 1998 and 2011 that investigated the efficacy of intralesional cidofovir reported at least one instance of disease remission (8). However, since most of these studies are uncontrolled case studies, it is unclear whether the benefits are caused by Cidofovir, or are part of the natural course of the disease. A randomized control trial found that there were no statistically significant differences in papilloma severity or Health-Related Quality of Life between a control group, treated with surgical interventions as needed and intralesional injections of saline, and the experimental group, treated with surgery and intralesional Cidofovir, after 12 months of treatment (9). Both groups experienced a significant decrease in the severity of the disease over the course of the year, indicating that the disease may regress over time on its own (9). Another review that looked at 27 studies of Cidofovir administration, all published before 2011, found that the average complete remission rate with cidofovir was 37% in pediatric studies (10). There have also been some severe side-effects associated with cidofovir, as a review found that 1.7 and 1% of 447 RRP patients experienced malignant transformation and nephrotoxicity, respectively (8). Other side effects include cutaneous rash, headache, and vocal cord scarring (11). Given these potential complications and the lack of an accepted protocol for dosage or frequency of administration, this drug is not a reliable adjuvant (4).

Cidofovir has also been administered as a systemic rather than local treatment for RRP cases that are complicated by lung disease. Four single case studies have shown that intravenous cidofovir led to disease remission, which is not the normal progression of the disease with lung involvement (11). All these studies employed hyperhydration and probenecid, a uric acid reducer, to reduce the chances of nephrotoxicity (11). Only one patient experienced side-effects, partial alopecia and leukopenia, from a combination therapy of cidofovir and interferon (12). The doses of both drugs were reduced in response. Eighteen months after the end of treatment, the patient had only required surgery for RRP once, and her lung disease had stabilized (12).

A novel method of Cidofovir delivery is inhalation. Few studies have investigated the efficacy of this method, but it may be promising for patients who have not responded to other forms of treatment, like a 4-month-old boy who, despite biweekly microdebridements and intralesional Cidofovir and intravenous interferon α injections, experienced worsening symptoms (13). Within 6 weeks of 40 mg of nebulized Cidofovir daily, 12 days on and 2 days off, the time between debridements increased (13). Six months later, the patient did not show any symptoms (13). To summarize, although Cidofovir in various administration forms remains widely used, its treatment results at best are mixed, and its potential for side effects are somewhat limiting.

## Photodynamic Therapy

Photodynamic therapy (PDT) involves introducing a light sensitive substance, called a photo-sensitizer, orally or by injection, into diseased tissue and activating the substance with laser light to induce necrosis. One of the major benefits of PDT is the ability to destroy tumors without affecting surrounding tissue (4). Case studies have shown significantly slower rates of papilloma regrowth soon after PDT, administered as an adjuvant or standalone therapy, and in some cases, the absence of disease after several weeks (14, 15). There have been only a few randomized control trials to investigate the impact of PDT, but the most recent found that the disease recurred after 3–5 years, likely because it generates its impact through a short-term immune response (16). Further research should be conducted to ascertain its efficacy and safety.

## Indole-3-Carbinol

Found in high concentrations in cruciferous vegetables like broccoli and cabbage, indole-3-carbinol modifies estrogen metabolism to alter cellular proliferation and DNA synthesis. The most recent clinical trial found that, after taking indole-3-carbinol twice a day, with a 200 mg dose for adults and weight-determined doses for children, for an average of 50.2 months, 70% of subjects experienced either a complete or partial response (17). Of the pediatric patients, one experienced a complete response, three a partial response, and five no response at all (17). It is unclear why adult patients responded better to indole-3-carbinol than pediatric patients. The less aggressive nature of adult-onset RRP may be a factor. Given the unpromising results in the pediatric population, there has not been much more research on treating juvenile onset RRP with of indole-3-carbinol.

## Celecoxib

Celecoxib is an anti-inflammatory drug that inhibits cyclooxygenase-2 (COX-2), an enzyme that leads to inflammation and pain, and is commonly used to treat arthritis. *In-vitro* studies have shown that papilloma cells overexpress COX-2 as a result of enhanced Epidermal growth factor receptor (EGFR) signaling, and that this activity is important to their growth (18). A combination therapy of celecoxib and erlotinib, a EGFR kinase inhibitor, helped control progressive RRP in a 58-year-old man a by slowing papilloma growth, rendering further surgery unnecessary (19). An ongoing randomized control trial sponsored by Northwell Health aims to determine whether celecoxib can decrease the rate of recurrence of papillomas in both adult and pediatric patients (4).

## Anti-Reflux Medication

Case studies have shown that treating gastroesophageal reflux disease (GERD) with anti- reflux medication in patients with juvenile-onset RRP can help slow the rate of papilloma regrowth (20, 21). In one study, patients who failed to comply with GERD treatment experienced recurrence (20). A retrospective chart review also showed that pediatric patients treated for RRP who were not treated for reflux were significantly more likely to develop laryngeal webs after the surgical removal of papillomas (22). While there is inconclusive evidence that GERD aggravates RRP, managing it makes sense for patients who have clinical presentation of GERD and complicated, progressive cases of RRP (23).

## Heat Shock Protein

Among the 700 children with RRP managed by 74 pediatric otolaryngologists, who were surveyed on their use of adjuvant treatment, 11 patients received HSP-E7, a recombinant fusion protein comprised of heat shock protein 65 (Hsp65) and the E7 protein of HPV type 16 (1). HSP-E7 has been investigated as a treatment in several diseases that stem from HPV, including genital warts and intraepithelial neoplasia (24). Evidence suggests that it may be reactive against more HPV strains than just HPV 16 (24). In an open-label trial of 27 pediatric RRP patients, the median interval between surgeries following treatment of HSP-E7 was significantly prolonged compared to the pre-treatment median (24). There were few complications, only mild-to-moderate injection site reactions. While there are no ongoing clinical trials to investigate the efficacy of Hsp-E7, it is a promising treatment.

## HPV Vaccine

A few case reports and studies have documented the use of the HPV vaccine as an adjuvant therapy. There are currently three approved HPV vaccines: the bivalent Cervarix, tetravalent Gardasil, and nonavalent Gardasil 9. The tetravalent vaccine acts targets HPV-6, HPV-11, HPV-16, HPV-18. A recent systematic review and meta-analysis found that the mean duration between surgeries in 63 juvenile and adult RRP patients significantly increased after HPV vaccination, from 7 to 34 months on average (25). The study found no significant differences by age of RRP onset (25). Other case studies not included in the meta-analysis have also shown Gardasil's efficacy in pediatric patients, as it increases the time between surgeries significantly or in some cases, causes complete remission (26, 27).

Intramuscular HPV vaccination might be more efficacious than previously identified, more common treatments like intralesional cidofovir. A retrospective case study that followed juvenile and adult-onset RRP patients for 22 years found that only two of the 13 patients treated with surgery and Gardasil relapsed, whereas all control patients, treated with surgery and Cidofovir, experienced papilloma regrowth (28). The average length of time to disease recurrence was also significantly longer in treated patients than controls (28).

One of the most promising aspects of HPV as adjuvant therapy is that it does not have any severe side effects. Additionally, with increasing vaccination rates, the incidence of juvenile RRP may decrease overall, as this form of the disease is commonly acquired when a baby is exposed to genital warts caused by the HPV 6 or 11 virus during childbirth (4).

## Mumps Vaccine

There have been very few studies on the efficacy of the mumps vaccine as an adjuvant therapy, although current research is promising. A case study found that remission was induced in nine of 11 pediatric patients treated with intralesional injections of the vaccine at 3- to 12-week intervals along with laser surgery (29). A retrospective study comparing Cidofovir and the measles, mumps, and rubella (MMR) vaccine as adjuvant therapies in a pediatric population found that there were no significant differences between those children treated with intralesional cidofovir and MMR injections after debridements (30).

## Bevacizumab

Bevacizumab is human monoclonal antibody that binds to and prevents the interaction of vascular endothelial growth factor (VEGF) with receptors. VEGF activity has a role in RRP development, as *in vitro* studies have shown strong expression of VEGF-A in papilloma epithelium and the expression of VEGFR-1 and VEGFR-2 messenger RNAs in underlying vascular endothelial cells (31).

The first studies looking at bevacizumab as a treatment for RRP investigated the benefit of intralesional injections as an adjuvant therapy to surgery. A study of three patients between 3 and 6 years-of-age with severe RRP (i.e., with at least four procedures per year) found that all patients experienced increased time between surgical debridement and pulsed KTP laser treatments and less severe RRP several weeks after the termination of bevacizumab treatment compared to before (32). They also showed improved voice-related quality of life (32).

Aside from case reports, case control studies have also been conducted to investigate the efficacy of bevacizumab as adjuvant therapy. After 532 nm KTP laser treatment followed by sublesional bevacizumab injections in the more diseased vocal fold, and saline injections in the other fold, 16 of the 20 adult patients with bilateral vocal fold RRP had fewer papilloma in the treated fold, as determined by endoscopic imaging (33). Three of the 20 patients had no disease in either fold, and none of the patients experienced any complications from the treatment (33).

Based on these promising results, a larger study looking at the efficacy of bevacizumab was conducted in a pediatric population. In 10 children between 18 months and 18 years with progressive, non-responsive RRP, three intralesional bevacizumab injections of 2.5 mg/ml 2–3 weeks apart, along with laser therapy, increased the median length of time between surgical procedures, decreased the median number of procedures per year, and improved voice-related quality of life (3). One of the limitations of the study is that the dosing is an estimation based on doses in pediatric ophthalmology. The amount injected into paillomas was also not the same for every patient, as it varied based on severity of disease.

Some studies have tried to determine optimal dosages. Another study looking at nine pediatric patients with juvenile onset RRP also found that, after a series of five subepithelial injections administered at 4–6 week intervals, with a mean dose of 14.25 mg, together with KTP laser ablation, all nine patients (with a median age of 8 years) experienced increased time interval between injections (34). These results indicate that showed that high-dose bevacizumab treatment does not have any complications and may be highly effective.

Following evidence that showed the efficacy of intralesional bevacizumab, research turned to address systemic bevacizumab, that is an especially promising treatment for patients with complex diagnoses. The first report of intravenous administration showed that, with a median of 6 courses in doses of 5, 10, 15 mg/kg, all treated patients with progressive RRP showed papilloma regression (35). The patients included four cases of adult onset RRP and one juvenile onset. The five patients collectively underwent 18 surgical interventions the year before bevacizumab, but only the adult case required surgery after treatment because of a malignant transformation the following year (35).

Other single case reports have corroborated the efficacy of systemic bevacizumab without finding complications, although most of these are in adult patients. In a 42 year old man with severe tracheal RRP, a low dose of 5 mg/kg, increased to 10 mg/kg of bevacizumab over time, helped achieve disease regression 3 months after treatment cessation (36). A 12-month follow-up revealed the patient remained disease free. Six courses of systemic bevacizumab treatment of 5 mg/kg every 2 weeks for a 87-year-old patient who had not responded to intralesional cidofovir, an endobronchial stent, or HPV vaccination, also helped achieve a significant decrease in the mass of the lobe and a patent bronchus (37). After two courses of 10 mg/kg intravenous injections every 2 weeks, a 63-year-old with severe RRP showed less lung involvement and no evidence of tracheal obstruction (37).

The only published case report in children is of a 12-year-old female with progressive laryngotracheal papillomatosis and lung involvement who, after 3 months of initiating systemic bevacizumab treatment, showed a partial response in the larynx and an almost complete response in the trachea (38). After 5 months, the patient no longer showed lung involvement. These results are even more impressive given that, over a 10-year span, the patient had been initiated on Gardasil, interferon, celecoxib, anti-reflux medication, zithromycin, and propranolol, and not responded to any (38).

Systemic bevacizumab's potential as treatment for the most aggressive forms of RRP is best summarized by the results of an electronic survey of the RRP Task Force of the American Society of Pediatric Otolaryngology, American Broncho-Esophagological Association, and physicians who have treated RRP with systemic bevacizumab (39). The 11 completed surveys obtained from nine medical centers showed that most of the patients treated with bevacizumab had a long history of juvenile onset RRP and had failed to show sustained responses to cidofovir, interferon, and celecoxib. Physicians gave treatment in the range of 5–10 mg/kg per dose. Seven of the eight patients treated had a partial response and one showed a complete response to treatment (39). All patients experienced increased time between surgeries, now on the order of months. In three of the four patients with lung involvement, three showed the improvement or resolution of pulmonary papilloma and one showed the stabilization of the disease. Only two patients experienced minor complications, hemoptysis, and proteinuria.

To ascertain the causal effect of bevacizumab, determine appropriate doses, and identify any potential complications, randomized control trials and larger case control studies must be conducted. Moreover, protocols to define duration of treatment, while controlling for any potential rebound effects need to be elaborated.

## Conclusions

Although an optimal, universally effective medical adjuvant therapy for RRP has not been yet found, promising results from RRP vaccination as a therapeutic approach along with the usage of systemic bevacizumab offer hope for improved outcomes for these children in the coming years. Optimal protocols for treatment should be developed in the near future.

## Author Contributions

All authors listed have made a substantial, direct and intellectual contribution to the work, and approved it for publication.

### Conflict of Interest Statement

The authors declare that the research was conducted in the absence of any commercial or financial relationships that could be construed as a potential conflict of interest. The handling editor declared a past co-authorship with one of the authors DP.
